# Supplementary material for: Attitudes and Beliefs of Primary Care Physicians and Nurses in Spain Toward Vegan Diets
Source: Nutrients. 2024 Nov 21;16(23):3992. doi: 10.3390/nu16233992 (PMC11643786; doi:10.3390/nu16233992)
Supplement: Supplementary file 1 [file nutrients-16-03992-s001.zip › nutrients-3291860-supplementary.pdf]

Questionnaire on Diet Free from Animal Products

This questionnaire is part of a research study conducted by the University of Cádiz. The objectives of this study are to identify the stance of Health Professionals towards vegan diets (free from animal products) and to determine their attitudes and beliefs regarding these diets.

The questionnaire is anonymous and does not collect any identifying data, ensuring that your responses will not be associated with you personally. The data collected will be used solely for this research study.

Completing the questionnaire takes no more than 5 minutes. Although the questionnaire can be completed multiple times by the same person, we kindly ask that you only complete it once. Before beginning, you will need to check the box for informed consent to allow us to analyse your responses. To finalise, please also check the last item regarding privacy in the use of Google Forms.

It would be very helpful if, once completed, you could share it with other health professionals you know to help increase the number of participants.

Thank you very much for your collaboration!

General Information

1. **Informed Consent**
    - I give my consent to participate in this questionnaire. I have been informed about the objectives of this study. Participation is entirely voluntary and anonymous. Personal data will be handled according to the following regulations: Article d, Section 2, of the additional provision seventeen, of Organic Law 3/2018, of 5 December, on Personal Data Protection and the guarantee of digital rights, regarding the use of pseudonymised personal data for health research, and the REGULATION (EU) 2016/679 OF THE EUROPEAN PARLIAMENT AND OF THE COUNCIL of 27 April 2016 on the protection of individuals with regard to the processing of personal data and the free movement of such data, repealing Directive 95/46/EC (General Data Protection Regulation).
2. **Age (in years)**
    - 20–30
    - 31–40
    - 41–50
    - 51–60
    - ≥ 61
3. **Gender**
    - Female
    - Male
4. **Profession**
    - Physician

- Nurse

5. **Location of Workplace**
    - Urban area (More than 50,000 inhabitants)
    - Semi-rural area (between 5,000 and 49,999 inhabitants)
    - Rural area (up to 4,999 inhabitants)
6. **Do you have specific training in nutrition?**
    - Yes, I have a university degree or postgraduate qualification.
    - Yes, I have completed one or more continuing education courses in nutrition.
    - No, I do not have specific training in nutrition.
7. **Which option best describes your current dietary habits?**
    - Omnivore (regular meat consumption)
    - Flexitarian (occasionally abstain from meat but do not completely renounce it)
    - Vegetarian/Vegan (lacto-ovo vegetarian, vegetarian, or vegan)

Environmental Stance

8. **Please indicate your level of agreement with the following statements:**
(Mark one option per row)
    - **Livestock farming has a high environmental impact.**
    - **Livestock farming is one of the causes of global warming.**

        - Strongly Disagree
        - Disagree
        - Neither Agree nor Disagree
        - Agree
        - Strongly Agree

Dietary Position

9. **Please indicate your level of agreement with the following statements:**
(Mark one option per row)

    - **I believe that a vegan diet (free from animal products*) is not suitable for pregnant women, breastfeeding mothers, children, or the elderly, even when it is followed with adequate nutritional knowledge and supplemented with vitamin B12.**
    - **Following a vegan diet (free from animal products*) is more expensive.**
    - **I would be willing to replace natural meat with lab-grown meat when it becomes commercially available.**

        - Strongly Disagree
        - Disagree
        - Neither Agree nor Disagree

- Agree
- Strongly Agree

** A diet completely free from animal products, including meat, dairy, eggs, and any other animal-derived ingredients.*

Professional Position on Vegan Diets

10. **Please indicate your level of agreement with the following statements:**
    (Mark one option per row)

- **If a healthy adult patient told me they follow a vegan diet, I would try to dissuade them from continuing it.**
  - Strongly Disagree
  - Disagree
  - Neither Agree nor Disagree
  - Agree
  - Strongly Agree

Nutritional Adequacy of a Vegan Diet

11. **Please indicate your level of agreement with the following statements:**
    (Mark one option per row)

- **Following a vegan diet with adequate nutritional knowledge and vitamin B12 supplementation does not provide the necessary macronutrients and micronutrients.**
  - **It may lead to deficiencies in recommended protein intake.**
  - **It may lead to deficiencies in recommended iron intake.**
  - **It may lead to deficiencies in recommended calcium intake.**

  - Strongly Disagree
  - Disagree
  - Neither Agree nor Disagree
  - Agree
  - Strongly Agree

---

12. **Privacy Policy**

- I accept the privacy policy: https://policies.google.com/privacy?hl=es

---

Cuestionario sobre Dieta Libre de Productos de Origen Animal

Este cuestionario es parte de un estudio de investigación realizado por la Universidad de Cádiz. Los objetivos de este estudio son identificar la postura de los profesionales de la salud hacia las dietas veganas (libres de productos de origen animal) y determinar sus actitudes y creencias sobre estas dietas.

El cuestionario es anónimo y no recopila datos identificativos, garantizando que sus respuestas no estarán asociadas a su identidad. Los datos recopilados se utilizarán únicamente para este estudio de investigación.

Completar el cuestionario no le tomará más de 5 minutos. Aunque la misma persona puede completarlo varias veces, le pedimos amablemente que lo haga solo una vez. Antes de comenzar, deberá marcar la casilla de consentimiento informado para permitirnos analizar sus respuestas. Para finalizar, también debe marcar el último elemento relativo a la política de privacidad de Google Forms.

Nos sería de gran ayuda si, una vez completado, pudiera compartirlo con otros profesionales de la salud que conozca para aumentar el número de participantes.

¡Muchas gracias por su colaboración!

Información General

Consentimiento Informado
Doy mi consentimiento para participar en este cuestionario. He sido informado sobre los objetivos de este estudio. La participación es totalmente voluntaria y anónima. Los datos personales se tratarán de acuerdo con las siguientes normativas: Artículo d, Sección 2, de la disposición adicional decimoséptima de la Ley Orgánica 3/2018, de 5 de diciembre, de Protección de Datos Personales y garantía de derechos digitales, respecto al uso de datos personales seudonimizados con fines de investigación en salud; y el REGLAMENTO (UE) 2016/679 DEL PARLAMENTO EUROPEO Y DEL CONSEJO de 27 de abril de 2016 sobre la protección de las personas físicas en lo que respecta al tratamiento de datos personales y a la libre circulación de estos datos, derogando la Directiva 95/46/CE (Reglamento general de protección de datos).

Edad (en años)

- 20–30
- 31–40
- 41–50
- 51–60
- ≥ 61

Sexo

- Femenino
- Masculino

Profesión

- Médico/a
- Enfermero/a

Localización del Centro de Trabajo

- Zona urbana (más de 50,000 habitantes)
- Zona semi-rural (entre 5,000 y 49,999 habitantes)
- Zona rural (hasta 4,999 habitantes)

¿Tiene formación específica en nutrición?

- Sí, tengo un título universitario o de posgrado.
- Sí, he realizado uno o varios cursos de formación continua en nutrición.
- No, no tengo formación específica en nutrición.

¿Qué opción describe mejor sus hábitos dietéticos actuales?

- Omnívoro (consumo regular de carne)
- Flexitariano (ocasionalmente me abstengo de carne, pero no la renuncio completamente)
- Vegetariano/Vegano (lacto-ovo vegetariano, vegetariano o vegano)

Postura Ambiental

Por favor, indique su nivel de acuerdo con las siguientes afirmaciones:
(Marque una opción por fila)

- **La ganadería tiene un alto impacto ambiental.**
- **La ganadería es una de las causas del calentamiento global.**
    - Totalmente en desacuerdo
    - En desacuerdo
    - Ni de acuerdo ni en desacuerdo
    - De acuerdo
    - Totalmente de acuerdo

Posición sobre la Dieta

Por favor, indique su nivel de acuerdo con las siguientes afirmaciones:
(Marque una opción por fila)

- **Considero que una dieta vegana (libre de productos de origen animal*) no es adecuada para mujeres embarazadas, madres lactantes, niños o ancianos, incluso cuando se sigue con los conocimientos nutricionales adecuados y se suplementa con vitamina B12.**

- **Seguir una dieta vegana (libre de productos de origen animal*) es más costoso.**
- **Estaría dispuesto a reemplazar la carne natural por carne cultivada en laboratorio cuando esté disponible comercialmente.**
    - Totalmente en desacuerdo
    - En desacuerdo
    - Ni de acuerdo ni en desacuerdo
    - De acuerdo
    - Totalmente de acuerdo

* *Una dieta completamente libre de productos de origen animal, incluyendo carne, lácteos, huevos y cualquier otro ingrediente derivado de animales.*

Posición Profesional sobre las Dietas Veganas

Por favor, indique su nivel de acuerdo con las siguientes afirmaciones:
(Marque una opción por fila)

- **Si un paciente adulto sano me dijera que sigue una dieta vegana, intentaría disuadirle de continuarla.**
    - Totalmente en desacuerdo
    - En desacuerdo
    - Ni de acuerdo ni en desacuerdo
    - De acuerdo
    - Totalmente de acuerdo

Adecuación Nutricional de una Dieta Vegana

Por favor, indique su nivel de acuerdo con las siguientes afirmaciones:
(Marque una opción por fila)

- **Seguir una dieta vegana con conocimientos nutricionales adecuados y suplementada con vitamina B12 no proporciona los macronutrientes y micronutrientes necesarios.**
- **Puede llevar a deficiencias en la ingesta recomendada de proteínas.**
- **Puede llevar a deficiencias en la ingesta recomendada de hierro.**
- **Puede llevar a deficiencias en la ingesta recomendada de calcio.**
    - Totalmente en desacuerdo
    - En desacuerdo
    - Ni de acuerdo ni en desacuerdo
    - De acuerdo
    - Totalmente de acuerdo

Política de Privacidad

- Acepto la política de privacidad: https://policies.google.com/privacy?hl=es
